# Joint analysis of PK and immunogenicity outcomes using factorization model − a powerful approach for PK similarity study

**DOI:** 10.1186/s12874-022-01742-2

**Published:** 2022-10-08

**Authors:** Halimu N. Haliduola, Fausto Berti, Heimo Stroissnig, Eric Guenzi, Hendrik Otto, Abid Sattar, Ulrich Mansmann

**Affiliations:** 1 Alvotech Germany GmbH, Jülich, Germany; 2 Alvotech Swiss AG, Zürich, Switzerland; 3 Alvotech UK LTD, London, UK; 4 Institute for Medical Information Processing, Biometry and Epidemiology – IBE, LMU Munich, Munich, Germany

**Keywords:** Biosimilars, PK similarity, Immunogenicity, Factorization model, Bioequivalence

## Abstract

Biological products, whether they are innovator products or biosimilars, can incite an immunogenic response ensuing in the development of anti-drug antibodies (ADA). The presence of ADA’s often affects the drug clearance, resulting in an increase in the variability of pharmacokinetic (PK) analysis and challenges in the design and analysis of PK similarity studies. Immunogenic response is a complex process which may be manifested by product and non-product-related factors. Potential imbalances in non-product-related factors between treatment groups may lead to differences in antibodies formation and thus in PK outcome. The current standard statistical approaches dismiss any associations between immunogenicity and PK outcomes. However, we consider PK and immunogenicity as the two correlated outcomes of the study treatment. In this research, we propose a factorization model for the simultaneous analysis of PK parameters (normal variable after taking log-transformation) and immunogenic response subgroup (binary variable). The central principle of the factorization model is to describe the likelihood function as the product of the marginal distribution of one outcome and the conditional distribution of the second outcome given the previous one. Factorization model captures the additional information contained in the correlation between the outcomes, it is more efficient than models that ignore potential dependencies between the outcomes. In our context, factorization model accounts for variability in PK data by considering the influence of immunogenicity. Based on our simulation studies, the factorization model provides more accurate and efficient estimates of the treatment effect in the PK data by taking into account the impact of immunogenicity. These findings are supported by two PK similarity clinical studies with a highly immunogenic biologic.

**Supplementary Information:**

The online version contains supplementary material available at 10.1186/s12874-022-01742-2.

## Introduction

In pharmacokinetic (PK) similarity studies, the primary analysis to assess equivalence between Test (T) and Reference (R) products is based on the average equivalence approach to compare PK parameters such as area under the curve (AUC) and peak concentration (C_max_) [1]. Analysis of variance (ANOVA) or analysis of covariance (ANCOVA) is commonly used statistical method to assess the equivalence of T and R products. These models use log-transformed PK parameters as outcome variables and treatment group and relevant covariates as fixed effects. The average equivalence approach involves calculating 90% confidence intervals (CIs) for the geometric mean ratio (GMR) of the PK parameters between T and R products. To establish PK similarity, the calculated 90% CIs should fall within an acceptable margin of [0.80, 1.25] for all primary PK parameters.

**Fig. 1:**
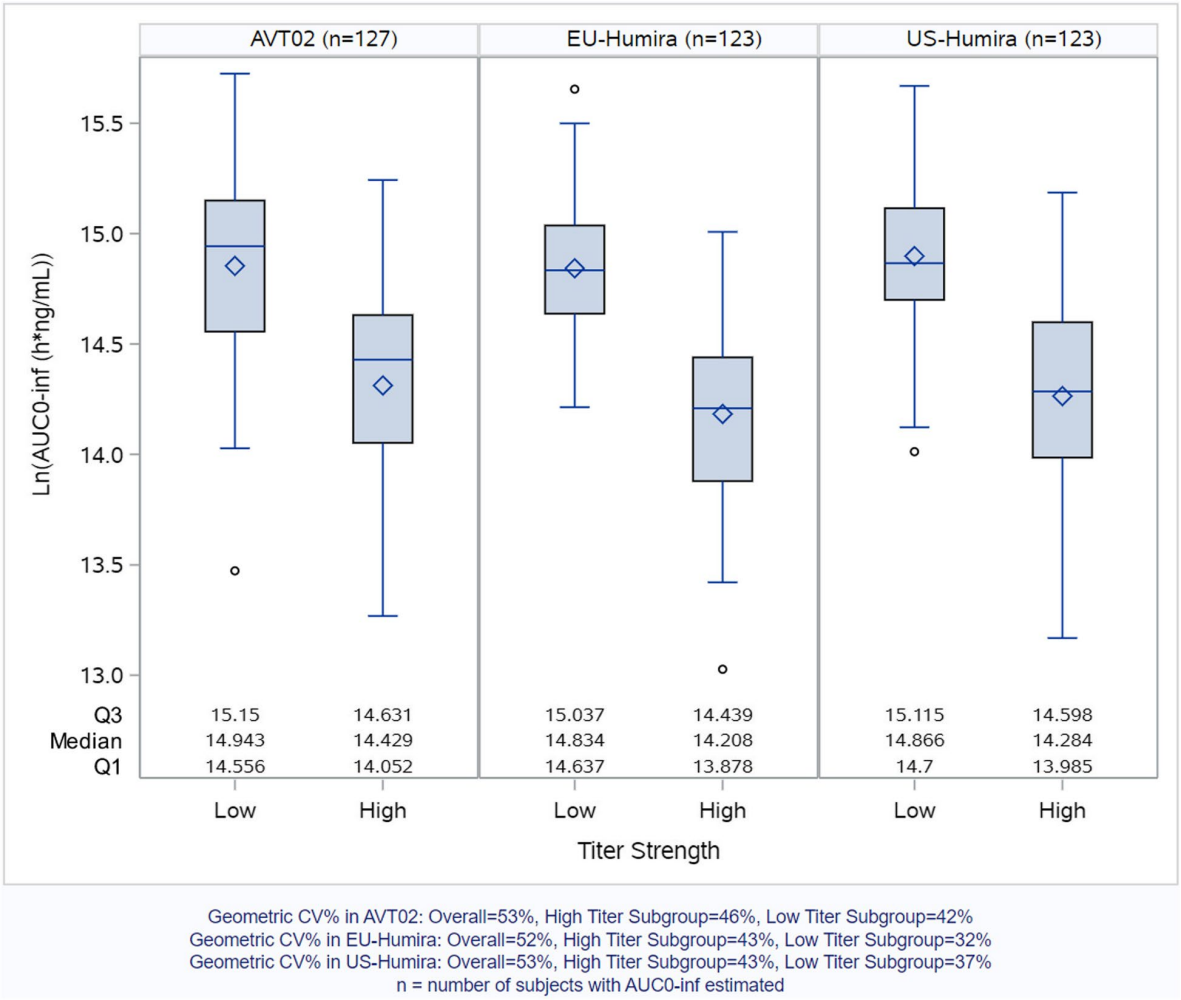
Real data from AVT02-GL-101 study. Distribution of AUC_0-inf_ by treatment group and immunogenic subgroup (“high titer level” versus “low or zero titer level”)

**Fig. 2:**
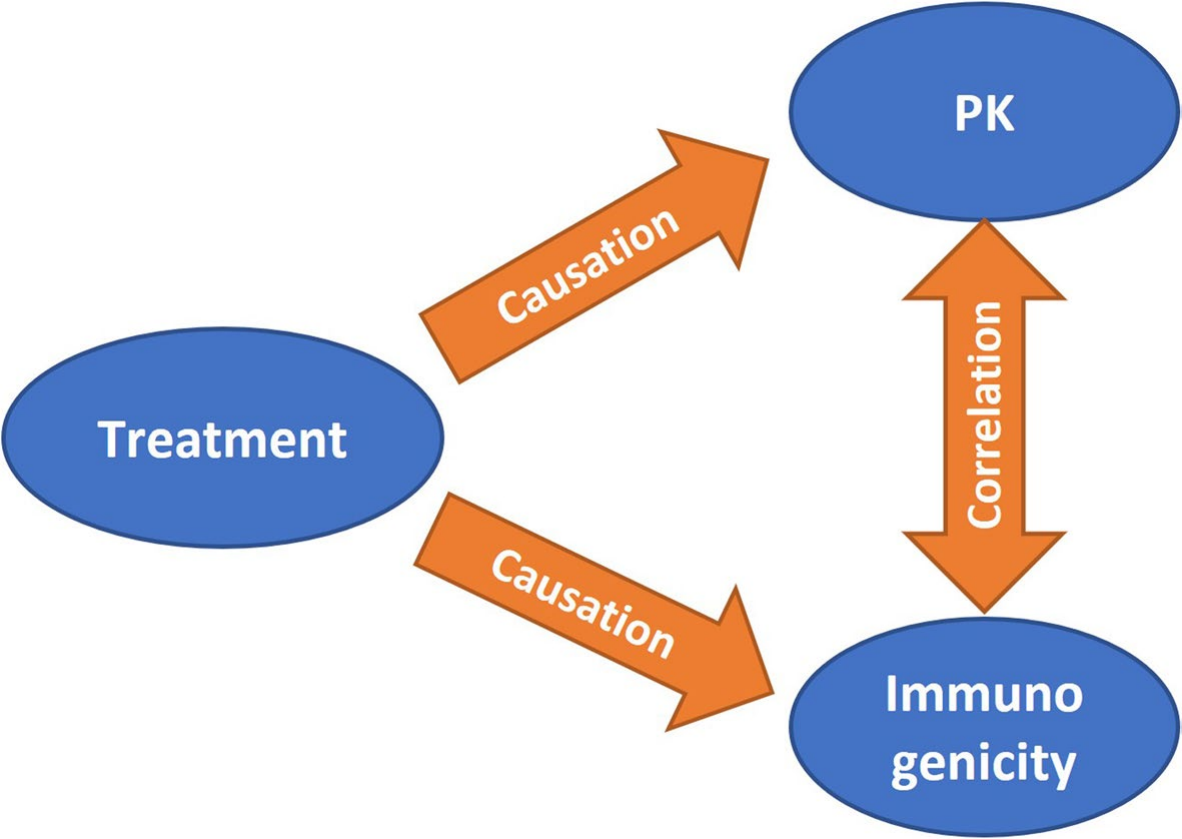
Diagram for the relationship between treatment, PK and immunogenicity outcomes

Many biological products have a long half-life and elicit immunogenic response, therefore, parallel group designs are often used in PK similarity studies. The development of immunogenicity poses many challenges in the design and analysis of PK similarity studies:

- First, the development of anti-drug antibodies (ADA) may affect drug clearance and thus PK profiles [2]. For example, in adalimumab PK similarity studies, it is common for ADA-positive subjects to have lower PK exposure in terms of AUC_0-inf_ and AUC_0-last_ compared to ADA-negative subjects [3, 4]. In Fig. 1, we present an example of the distribution of AUC_0-inf_ in the AVT02-GL-101 study (a pivotal PK similarity study of adalimumab biosimilar [5]). Within each treatment group, the distribution of AUC_0-inf_ differed substantially in terms of central tendency between immunogenic subgroups (i.e., lower PK exposure was observed in the high ADA titer level subgroup). In addition, as summarized in the footnote of Fig. 1, the geometric coefficient of variation (CV) of AUC_0-inf_ is greater in the high titer subgroup than in the low or zero titer subgroup in AVT02-GL-101 study. This is consistent with data reported in other adalimumab biosimilar studies, for example, Von Richter et al. [3] reported greater variability in AUC_0-inf_ and AUC_0-last_ in ADA-positive subjects than in ADA-negative subjects. Such difference in variability of PK parameters between immunogenic subgroups could be due to the diversity of immune responses in the study population (e.g., early versus late onset of ADA, different levels of immune response strength) leading to heterogeneity in PK profiles and therefore increased the overall variability of PK parameters. This is, at least partly, the reason for the relatively large sample sizes required in PK similarity studies for the highly immunogenic biologics.

- Secondly, the development of immunogenicity is a complex process. There are many product-related and non-product-related factors that may affect the immunogenicity of biologics [6]. Potential imbalances in non-product-related factors between treatment groups may lead to differences in immunogenicity and thus in PK outcomes. For example, Von Richter et al. [7] reported the immune response to the exact same biologic batch was markedly different between the two treatment groups (cross studies comparison) and hence affected the equivalence assessment. Currently available techniques do not allow one to predict which subjects will develop an immune response to a particular biologic and at what time during treatment [6]. Therefore, it is not possible to stratify subjects according to their propensity to develop an immune response at the time of randomization.
- In addition, development of immunogenicity is also an outcome of the treatment. The ADA response information (as a post-baseline variable) cannot be used in statistical models to take account for its effect on PK outcome. The ADA response variable is correlated with PK outcome variable, but it is not causal for PK outcome. See Fig. 2, an illustration of relationship between treatment, PK and immunogenicity outcomes. Apparently, treatment is the causal for these two correlated outcomes (PK and immunogenicity). In practice, subgroup analyses of PK parameters (e.g., ADA-positive/negative subgroups) are often used to assess the impact of immunogenicity on PK. However, subgroup analyses are not statistically powered and such marginal summary of PK data does not reveal the impact of immunogenicity on the overall treatment effect estimation.

- Finally, current standard statistical approaches that completely ignore potential associations between PK and immunogenicity outcomes (e.g., methods like ANOVA or ANCOVA as mentioned above) have their drawbacks. When potential dependencies between outcomes are ignored, the estimated covariate effects (e.g., treatment effects) in the model can be biased and inefficient because the model fails to capture the additional information contained in the correlation between outcomes [8, 9].

In this paper, we consider PK and immunogenicity as the two correlated outcomes of the study treatment. In order to efficiently estimate treatment effect in PK data considering the impact of immunogenicity, we investigate the factorization model [10] for the simultaneous analysis of log-transformed PK parameters (normal variable) and immunogenic response subgroup (binary variable). The main idea of the factorization model (a direct approach to connect the two outcomes) is to describe the likelihood function as the product of the marginal distribution of one outcome and the conditional distribution of the second outcome given the previous one. A convenient feature of the factorization model is that the model parameters maintain marginal interpretation in both outcomes. This is a very useful property because in our proposal the focus is on the analysis and interpretation of PK data considering the influence of immunogenicity. In addition, because the factorization model captures the additional information contained in the correlation between outcomes, it is more efficient than models that ignore potential dependencies between outcomes. In our context, factorization model accounts for variability in PK data by considering the influence of immunogenicity. This allows us to efficiently and accurately estimate the treatment effect in the PK parameters by considering the impact of immunogenicity.

In light of the “evidence-based computational statistics” [11, 12], we evaluate the factorization model in the practically relevant simulation studies and exemplify in real data sets from PK similarity clinical trials. We perform comprehensive simulations to fully investigate the operating characteristics of the factorization model, all relevant factors are taken into account and all possible combinations of those factors are thoroughly evaluated. Based on the simulation studies, factorization model provides more accurate and efficient estimates of the treatment effect in the PK data by taking into account the impact of immunogenicity. These findings are supported by the real data from two PK similarity clinical studies with highly immunogenic biologics.

## Methods

As mentioned above, we consider PK and immunogenicity as the two correlated outcomes of the treatment. Let *y*_*ci*_ denote the continuous outcome (normal distributed dependent variable, e.g., log-transformed PK parameter), *y*_*bi*_ denote the binary outcome (binomial distributed dependent variable, e.g., ADA response: 1= ADA positive, 0= ADA negative; or ADA titer subgroup: 1= high titer level, 0= zero or low titer level) for the *i*^*th*^ subject, *y*_*bi*_ follow a binomial distribution,

1 $$P\left({y}_{bi}=1\right)=p,P\left({y}_{bi}=0\right)=q,0<p<1,p+q=1.$$

The conditional distribution of (*y*_*c*_ |*y*_*b*_ = 1) and (*y*_*c*_ |*y*_*b*_ = 0) are assumed to be *N*(*μ*_1_, *σ*_1_) and *N*(*μ*_0_, *σ*_0_), respectively. Therefore, *y*_*ci*_ follow a mixed distribution with the function [13],

2 $$F\left({y}_c\right)=p{F}_1\left({y}_c\right)+q{F}_0\left({y}_c\right),$$

where

3 $${F}_j\left({y}_c\right)=p\left({y}_c\le y\ |\ {y}_b=j\right)={\int}_{-\infty}^y\frac{1}{\sigma_j\sqrt{2\pi }}\ {e}^{-\left[\frac{{\left(z-{\mu}_j\right)}^2}{2{\sigma}_j^2}\right]}\ dz,j=0,1.$$

For the correlated continuous and binary outcomes in a cross-sectional setting, two general likelihood-based multivariate approaches have been developed: direct factorizing the joint distribution of the outcomes and introducing an unobserved (latent) variable to model the correlation among the multiple outcomes [14].

Since the immunogenicity has direct impact on the PK outcome (as discussed in section 1), we propose the factorization model [10] for analyzing PK and immunogenicity data jointly. Let *x*_*ci*_ and *x*_*bi*_ denote the covariate vectors associated with the outcome *y*_*ci*_ and *y*_*bi*_. We use a *probit* link (i.e., inverse of the *cdf* of the standard normal distribution) for the binary outcome and the *identity* link for the continuous outcome. In factorization model, the joint distribution of two outcomes is factorized into a marginal distribution of binary outcome and a conditional distribution of continuous outcome, given the binary outcome,

4 $$f\left({y}_b,{y}_c\right)=f\left({y}_b\right)f\left({y}_c|\ {y}_b\right).$$

The expected values of the outcomes given the covariate vectors *x*_*b*_ and *x*_*c*_ are defined as,

5 $$probit\left(E\left({y}_{bi}|\ {x}_{bi}\right)\right)= probit\left({\mu}_{bi}\right)={x}_{bi}^T{\beta}_b$$

and

6 $${y}_{ci} \mid {y}_{bi},{x}_{ci},{x}_{bi}={x}_{ci}^T{\beta}_c+\tau \left({y}_{bi}-{\mu}_{bi}\right)+{\epsilon}_{ci}$$

Where *β*_*b*_ *and β*_*c*_ are the marginal parameter vectors for covariate vectors *x*_*b*_ (i.e., independent variables for the immunogenicity outcome model) and *x*_*c*_ (i.e., independent variables for the PK outcome model), respectively, ${\epsilon}_{ci}\sim N\left(0,{\sigma}_c^2\right)$, ${\sigma}_c^2$ is the error variance of continuous outcome, and *τ* is the correlation of *y*_*ci*_ on *y*_*bi*_. Large absolute values of *τ* indicate a strong correlation between the two outcomes. If *τ* = 0, the two outcomes are independent given the covariates. The two models are linked by the product of correlation between the two outcomes and the residuals of binary variable.

The parameter estimates for the factorization model can be obtained by the commonly used maximum likelihood algorithm. The log-likelihood function under the factorization model is defined as,

7 $${\displaystyle \begin{array}{c}l\left({y}_b,{y}_c\right)=\mathit{\log}\prod\limits_{i=1}^nf\left({y}_{bi},{y}_{ci}\ |\ {x}_{bi},{x}_{ci}\right)=\mathit{\log}\prod\limits_{i=1}^nf\left({y}_{ci}|\ {y}_{bi},{x}_{ci},{x}_{bi}\right)\ f\left({y}_{bi}\ |\ {x}_{bi}\right)\kern0.5em \\ {}=\sum\limits_{i=1}^n\left(-\frac{1}{2}\log \left(2\pi {\sigma}_c^2\right)-\frac{1}{2{\sigma}_c^2}{\left({y}_{ci}-{\mu}_{ci}-\tau \left({y}_{bi}-\varphi \left({\mu}_{bi}\right)\right)\right)}^2\right)+\sum\limits_{i=1}^n\left({y}_{bi}\log \left(\varphi \left({\mu}_{bi}\right)\right)+\left(1-{y}_{bi}\right)\log \left(\varphi \left(1-{\mu}_{bi}\right)\right)\right)\ \end{array}}$$

where *φ*() is the *cdf* of the standard normal distribution, ${\mu}_{ci}={x}_{ci}^T{\beta}_c$, $probit\left({\mu}_{bi}\right)={x}_{bi}^T{\beta}_b$.

The correlation of outcomes that results from this model is [14],

8 $$Corr\left({y}_{bi},{y}_{ci}|{x}_{bi},{x}_{ci}\right)=\left\{\begin{array}{c}\frac{\mathit{\operatorname{sign}}\left(\tau \right)}{\sqrt{1+\frac{\sigma_c^2}{\tau^2\ Var\left({y}_{bi}|{x}_{bi}\right)}}}, if\ \tau \ne 0\\ {}0, if\ \tau =0\ \end{array}\right.$$

A convenient property of the factorization approach is that the model parameters maintain marginal interpretation in both regression equations. This is a very important and useful feature, as in our proposal, the focus is to analyze and interpret the PK data considering the impact of the immunogenicity. This will allow us to accurately estimate the treatment effect in the PK parameters by taking the impact of immunogenicity into account.

Another important feature of this model is the assumption regarding the distribution of *y*_*ci*_. Conditional on *y*_*bi*_ and the covariates (*x*_*bi*_, *x*_*ci*_), *y*_*ci*_ is assumed to be normally distributed, implying that the marginal distribution of *y*_*ci*_ is a mixture of two normal distributions. For a high correlation between the two outcomes, the marginal distribution of *y*_*ci*_ ∣ *x*_*bi*_, *x*_*ci*_ will in fact be bimodal. Therefore, the covariance of *y*_*ci*_ ∣ *x*_*bi*_, *x*_*ci*_ depends on *x*_*bi*_,

9 $$Var\left({y}_{ci}|{x}_{bi},{x}_{ci}\right)={\tau}^2\varphi \left({x}_{bi}^T{\beta}_b\right)\left(1-\varphi \left({x}_{bi}^T{\beta}_b\right)\right)+{\sigma}_c^2$$

This assumption is consistent with the actual distribution of PK parameters in immunogenic subgroups in PK similarity studies with highly immunogenic biologics. For example, as shown in Fig. 1, the distribution of AUC_0-inf_ differed substantially between immunogenic subgroups, i.e., within each treatment group it is a mixture of two normal distributions.

In addition, because the factorization model captures the additional information contained in the correlation between outcomes, it is more efficient than models that ignore potential dependencies between outcomes. In our context, factorization model accounts for variability in PK data by considering the influence of immunogenicity. This makes it more efficient than the standard analytical approaches like ANOVA or ANCOVA. It should be noted that the efficiency can be gained by adopting this approach only when the mean outcomes depend on different covariate sets, i.e., *x*_*b*_ and *x*_*c*_ are different [14]. Since the main purpose is to analyze and interpret the PK data considering the effect of immunogenicity, the main effect (i.e., treatment effect) should be included in the equation for *μ*_*ci*_ only, not in the model for binary outcome (otherwise the treatment effect may be diluted). It is also important to note that in our proposal, the aim is not to explain differences in PK data in terms of differences in immunogenicity between treatment groups, but rather to explain the overall variability of PK data by taking the influence of immunogenicity into account.

We use SAS Proc NLMIXED for the implementation of factorization model.

### Simulation study to evaluate performance of proposed method

#### Design of simulation study

We perform Monte Carlo simulation to investigate the operating characteristics of factorization model in the context of PK similarity study with the impact of immunogenicity. Simulation data were generated assuming a two-arm parallel designed PK similarity study with single dose administration. The correlated normal and binary data are generated simultaneously using the point-biserial correlation [15]. Suppose that *Y*_1_ and *Y*_2_ follow a bivariate normal distribution with a correlation of ${\rho}_{Y_1{Y}_2}$. If *Y*_1_ is dichotomized to produce *Y*_1*D*_, then the resulting correlation between *Y*_1*D*_ and *Y*_2_ can be given as point-biserial correlation ${\delta}_{Y_{1D}{Y}_2}$ [15]:

10 $${\delta}_{Y_{1D}{Y}_2}={\rho}_{Y_1{Y}_2}\left(\frac{h}{\sqrt{p\left(1-p\right)}}\right)$$

where *p* is the proportions of the observations above the point of dichotomization, and *h* is the ordinate (probability density function) of the normal curve at the same point. We use SAS Proc IML for the data generation.

Within each treatment group (T or R), given the correlation matrices, three correlated variables are generated simultaneously, including log-transformed PK parameter (normal distribution), ADA response binary variable (1= ADA positive, 0= ADA negative), and a baseline covariate (normal distribution) which is correlated to ADA response but independent from PK variable. The log-transformed PK parameter and ADA response are correlated. In order to fully investigate the operating characteristics of the factorization model, all relevant factors are considered in a simulation scenario and all possible combinations of these factors are evaluated.

To evaluate the statistical power and robustness of factorization model in the treatment effect estimation, we consider the following factors (36 scenarios in total for all possible combinations):

The marginal difference in log-transformed PK parameter between treatment group is *ln*(GMR (T/R) = 0.95).

- The geometric *CV* = 0.4 for the PK parameter in both treatment group.
- Data are generated for 130 subjects in each simulation study (which is the sample size that needed for ANOVA to maintain 80% power given the assumed GMR =0.95, geometric CV =0.4, *α* = 5%, and a standard equivalence margin of [0.80, 1.25]).
- In R group, ADA response rate (*P*_2_) is 20 % , 40 % *or* 60%; in T group, ADA response rate *P*_1_ ≥ *P*_2_, i.e., with a difference of 0%, 5 % *or* 10%. For instance, if *P*_2_ = 20%, then three different scenarios are considered for *P*_1_, i.e., *P*_1_ = 20 % , 25 % *or* 30%. It is worth noting that we assume the difference in ADA rates is due to non-product-related factors.
- The correlation coefficient of PK and ADA response is −0.3 or − 0.5.
- The correlation coefficient of baseline covariate and ADA response is 0.1 or 0.3. To be conservative on the model performance, relatively low correlation coefficients are considered for the covariate.

To evaluate the Type I error rate, we consider the following factors (6 scenarios in total for all possible combinations):

- The marginal difference in log-transformed PK parameter between treatment group is *ln*(GMR (T/R) = 0.80) (i.e., to see the error rate at the boundary of the standard equivalence margin).
- The geometric *CV* = 0.4 for the PK parameter in both treatment group.
- Data are generated for 200 subjects in each simulation study (i.e., a common PK similarity study size).
- ADA response rates are equal between T and R, i.e., *P*_1_ = *P*_2_ = 20 % , 40 % *or* 60%.
- The correlation coefficient of PK and ADA response is −0.3 or − 0.5.
- The correlation coefficient of baseline covariate and ADA response is 0.1. To be conservative on the model performance, low correlation strength is considered for the covariate.

For the exemplification of sample size determination, we consider the following factors (12 scenarios in total for all possible combinations):

The marginal difference in log-transformed PK parameter between treatment group is *ln*(GMR (T/R) = 0.95).

- The geometric *CV* = 0.3, 0.4, 0.5, or 0.6 for the PK parameter in both treatment group.
- ADA response rate is 50% for both T and R groups.
- The correlation coefficient of PK and ADA response is −0.3, − 0.5 or − 0.6.
- The correlation coefficient of baseline covariate and ADA response is 0.1. To be conservative on the model performance, low correlation strength is considered for the covariate.
- Data are generated for a number of subjects in each simulation study to maintain 80% power for factorization model using a standard equivalence margin of [0.80, 1.25] with *α* = 5%).

#### Measuring performance of the proposed methods

For each scenario, we analyze the log-transformed PK parameter data using the factorization model (with treatment group as factor in the linear model and using baseline continuous variable as covariate in the binary model) and the standard ANOVA model (with treatment group as factor). We perform the following measures to evaluate the operating characteristics of factorization model:

- Compare the statistical power of factorization model and ANOVA given different ADA response rates and correlation matrices.
- Compare the treatment effect in PK data (i.e., the actual GMR) estimated by factorization model and ANOVA given different ADA response rates and correlation matrices.
- Evaluate the Type I error rate of factorization model given different ADA response rates and correlation matrices.
- Compare the sample sizes that needed for the factorization model and ANOVA to maintain the same target statistical power given different correlation matrices and the variabilities of PK data.

#### Simulation results

##### Evaluation of statistical power

**Fig. 3:**
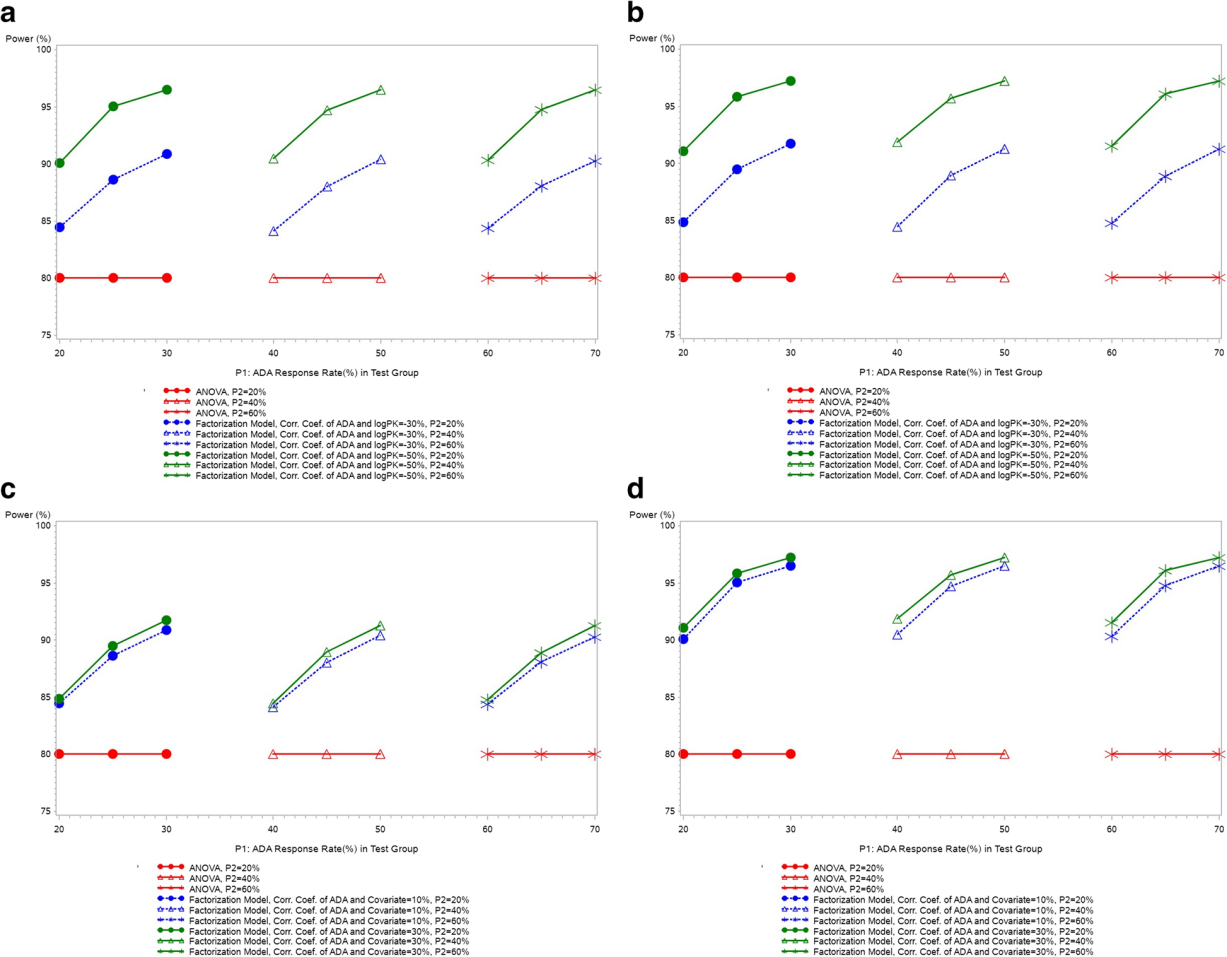
Comparison of power of factorization model and ANOVA. **a**: correlation coefficient of ADA and covariate = 0.1; **b**: correlation coefficient of ADA and covariate = 0.3; **c**: correlation coefficient of ADA and ln(PK) = − 0.3; **d**: correlation coefficient of ADA and ln(PK) = − 0.5. Note: (**c**) and (**d**) are different presentations of (**a**) and (**b**). In the PK parameter data generation, assumed GMR = 0.95, CV = 0.4 and total sample size = 130

The statistical power of factorization model and ANOVA for all simulated scenarios are plotted in Fig. 3. ANOVA maintains the target power of 80%, while factorization model improves the power significantly. The gained efficiency depends on: 1) the strength of correlation between PK and ADA data (strong factor, the higher correlation strength, the more variability explained by ADA data, the more efficiency gained); 2) the strength of correlation between ADA and covariate (weak factor, increasing correlation strength leads to slight increase in the power).

To fully investigate the operating characteristics of factorization model, in data generation step, we consider different scenarios for the ADA response rate, i.e., *P*_1_ ≥ *P*_2_, with a difference of 0%, 5 % *or* 10% between treatment groups. We assume the difference in ADA rate is due to non-product-related factors. In Fig. 3, the greater the difference in ADA rates, the higher the corresponding power. This is due to the fact that the difference in immunogenicity between the treatment groups leads to a difference in PK outcome (since these two outcomes are correlated) and there is more variability explained by the factorization model, thus increasing the power.

##### Evaluation of robustness of factorization model in treatment effect estimation

In data generation step, we set the marginal difference in log-transformed PK parameter between treatment group as *ln*(GMR (T/R) = 0.95). If the ADA response rate is higher in T than in R (the difference is assumed to be due to non-product-related factors in our simulation), considering the influence of immunogenicity on PK data, the actual “pure” treatment difference in PK data may be smaller than the marginal difference. In this case, the estimation of the marginal treatment difference is biased. An appropriate statistical analysis method should take such bias into account.

**Fig. 4:**
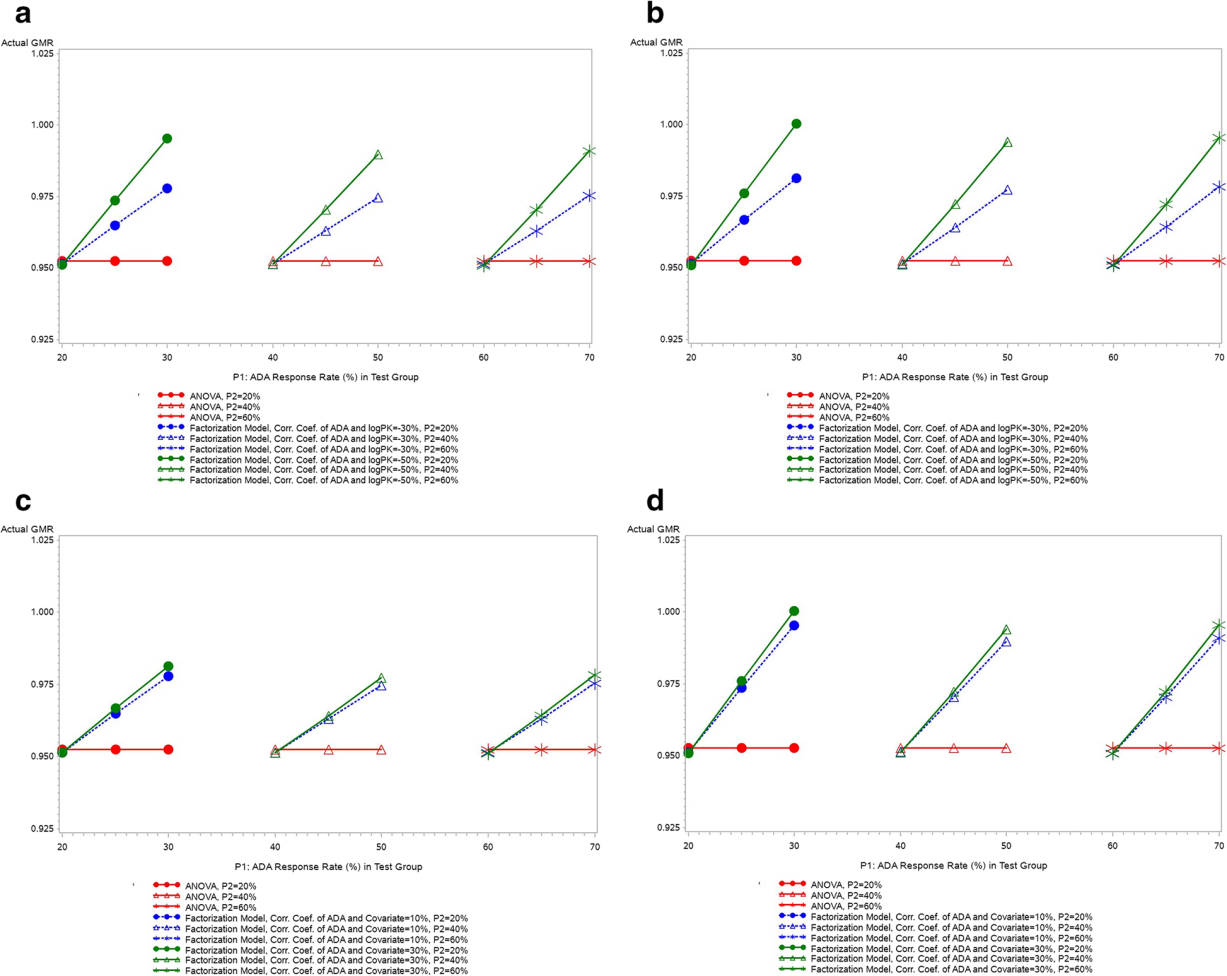
Comparison of actual GMR estimated from factorization model and ANOVA. **a**: correlation coefficient of ADA and covariate = 0.1; **b**: correlation coefficient of ADA and covariate = 0.3; **c**: correlation coefficient of ADA and ln(PK) = − 0.3; **d**: correlation coefficient of ADA and ln(PK) = − 0.5. Note: (**c**) and (**d**) are different presentations of (**a**) and (**b**). In the PK parameter data generation, assumed GMR = 0.95, CV = 0.4 and total sample size = 130

The actual GMRs estimated by factorization model and ANOVA are plotted for all simulated scenarios in Fig. 4. The actual GMRs are around 0.95 based on ANOVA as it estimates only the marginal treatment difference in PK data. While based on the factorization model, the actual GMR = 0.95 only when the ADA response rates are equal in two treatment groups; when the ADA response rates are different in two treatment groups, the actual GMRs are adjusted by factorization model to reflect the pure treatment effect in the PK data. The extent of such adjustment is proportional on the difference of ADA response rate between treatment group and also influenced by the strength of correlation between ADA and PK data (strong factor) and the strength of correlation between ADA and covariate (weak factor).

##### Evaluation of type I error rate of factorization model

We evaluate the Type I error rate of factorization model given different ADA response rates and correlation matrices. We assume equal ADA response rate between the treatment groups. As shown in Table 1, there is no inflation in the Type I error rate, i.e., the error rates are well controlled within 5% in all simulation scenarios.

**Table 1:** Type I error rate of factorization model by ADA response rate and correlation matrix

Scenario #	ADA response rate (%) ^**a**^	Correlation coefficient of PK and ADA	Type I error rate (%)
1	20%	−0.5	4.86%
2	40%	−0.5	4.85%
3	60%	−0.5	4.92%
4	20%	−0.3	4.98%
5	40%	−0.3	4.92%
6	60%	−0.3	4.86%

For PK parameter data generation, assumed GMR = 0.80 (at the boundary of the standard equivalence margin), assumed geometric CV = 0.4, correlation coefficient of ADA and covariate = 0.1

^a^ ADA response rates are equal for Test and Reference groups

##### Sample size determination examples

**Fig. 5:**
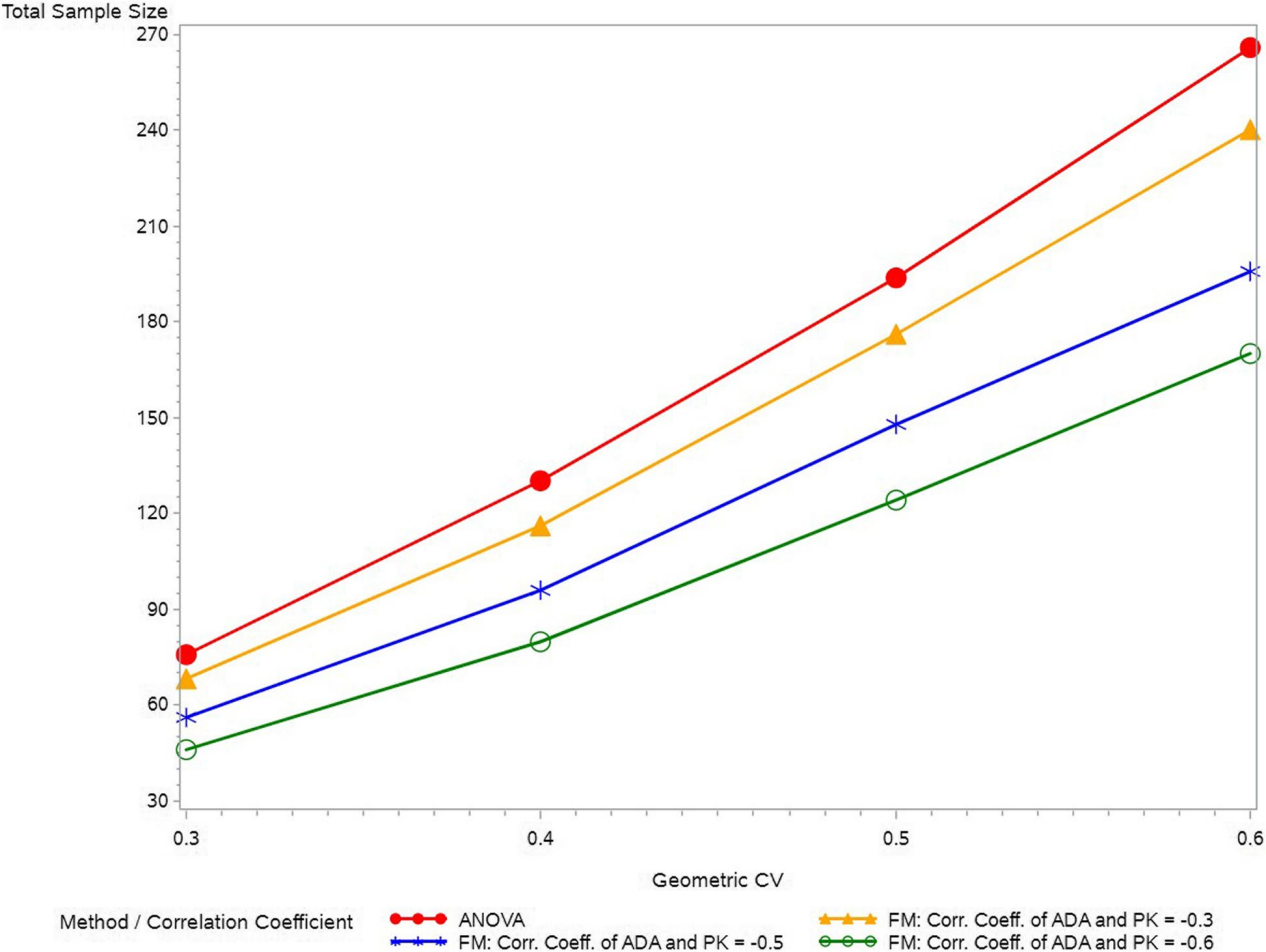
The sample sizes that needed for the factorization model and ANOVA to maintain the target power of 80% given different correlation matrices and CV in PK data. CV = coefficient of variance, ANOVA = analysis of variance, FM = factorization model

We compare the sample sizes (for a two-arm study with one comparison) that needed for the factorization model and ANOVA to maintain the same target statistical power given different correlation matrices and the variabilities of PK data. As shown in Fig. 5, the sample sizes that needed for the factorization model to maintain the same target power of 80% are considerably lower than the sample sizes that are needed for ANOVA. The higher correlation coefficient of the ADA and PK data, the more gain in the sample size.

### Real data example

We exemplify the proposed method in real data from PK similarity studies AVT02-GL-101 and AVT02-GL-102. AVT02-GL-101 is a three-arm pivotal study to demonstrate PK similarity of AVT02 (a biosimilar of adalimumab), EU-Humira and US-Humira following a single 40 mg subcutaneous injection in healthy adult volunteers [5]. AVT02-GL-102 is a two-arm study to demonstrate the PK similarity of AVT02 when administered via prefilled syringe (PFS) or autoinjector (AI) in healthy adult subjects (following a single 40 mg subcutaneous injection [16]). It is worth noting that the exact same AVT02 manufacturing batch was used in both the PFS and AI groups.

Since the majority of study subjects (> 90%) developed ADA at some point in time during the study period (Day 0 to Day 64) in both studies, subjects are split into immunogenic subgroups according to the ADA titer area under curve values (i.e., “high titer level” versus “low or zero titer level”) using the Classification and Regression Tree (CART) model [2]. In AVT02-GL-101 study, there is no meaningful difference in immunogenicity profiles between treatment groups. In the PK analysis set, there are 50.0% (64/128), 53.6% (67/125), and 54.3% (69/127) subjects are classified as “high titer level” in AVT02, EU-Humira, and US-Humira groups, respectively. The correlation coefficients of PK data (log-transformed) and immunogenic subgroup (1 = “high titer level”, 0 = “low or zero titer level”) are − 0.62, − 0.57 and − 0.24 for AUC_0-inf_, AUC_0-last_ and C_max_, respectively. In AVT02–102 study, there is slightly high titer strength in PFS group than in the AI group. In the PK analysis set, there are 65.7% (65/99) and 52.5% (52/99) subjects are classified as “high titer level” in PFS and AI groups, respectively. The correlation coefficients of PK data and immunogenic subgroup are − 0.59, − 0.54 and − 0.32 for AUC_0-inf_, AUC_0-last_ and C_max_, respectively. As mentioned, in AVT02-GL-102 study, the exact same manufacturing batch of AVT02 was used in both the PFS and AI groups, therefore, the difference in immunogenicity between the treatment groups can be considered to be due to unknown non-product-related factors.

**Fig. 6:**
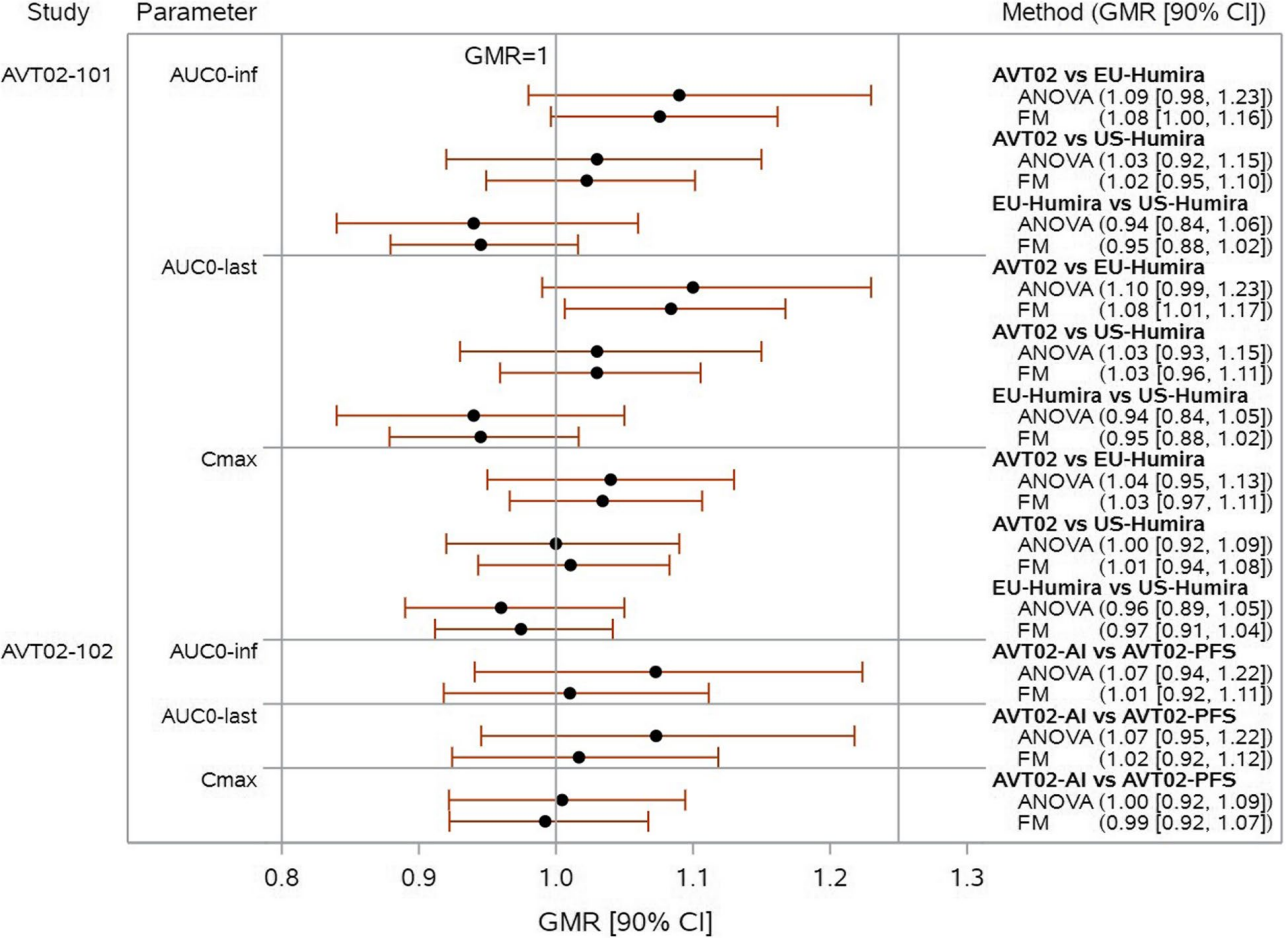
Analysis results of PK parameters using factorization model and ANOVA in AVT02-GL-101 and AVT02-GL-102 studies. GMR = geometric mean ratio, ANOVA = analysis of variance, FM = factorization model, PFS = prefilled syringe, AI = autoinjector

We analyze the PK parameter data from both studies using the factorization model (with treatment group and body weight as covariates for the linear model and with gender as covariate for the binary model) and the standard ANOVA model (with treatment group and body weight category as factors, i.e., using the planned analysis for the clinical study report). The analysis results are shown in Fig. 6.

- For the PK parameter C_max_ (which is less impacted by the immunogenic response as the onset of ADA mostly after the time point of C_max_), in both studies, the point estimates of GMR are similar for both models, the 90% CIs of the factorization model are slightly narrower than the ANOVA.
- For the PK parameters AUC_0-inf_ and AUC_0-last_ (which are substantially influenced by the immunogenic response), the 90% CIs of the factorization model are much narrower than the ANOVA in both studies. For the point estimates of GMR, different phenomena are observed:
  - ◦ In AVT02-GL-101 (where the immunogenicity profiles are balanced between treatment groups), the point estimates of GMR are similar in both models.
  - ◦ In AVT02-GL-102 (where the same AVT02 batch was used for both treatment groups but with slight difference in immunogenicity between groups due to unknown non-product-related factors), the point estimates of GMR in factorization model are much closer to 1.0 than the ANOVA (i.e., 1.01 vs. 1.07 for AUC_0-inf_ and 1.02 vs. 1.07 for AUC_0-last_). The ANOVA model estimates the marginal treatment difference in the PK data only and such estimation is biased due to influence of immunogenicity. This suggests that looking only at PK data and completely ignoring the impact of immunogenicity can be misleading. The treatment difference in PK data quantified by the ANOVA model is not only due to the treatment per se, it is confounded by the effect of immunogenicity. Factorization model takes into account the impact of immunogenicity and provides accurate treatment effect estimation on the PK data.

In addition, to evaluate the “extended” ANOVA/ANCOVA models that also include the factors that used in the binary model, additional analyses have been performed and results are provided in Additional file  2.

## Discussion

The development of immunogenicity poses many challenges in the design and analysis of PK similarity studies, as it increases the variability of PK data, and potential imbalances in non-product-related factors between treatment groups may lead to differences in immunogenicity and thus in PK outcomes. The current standard statistical approaches ignore potential associations between PK and immunogenicity outcomes. We consider PK and immunogenicity as the two correlated outcomes of the treatment and we propose factorization model for the simultaneous analysis of PK and immunogenicity data. Factorization model captures the additional information contained in the correlation between outcomes, provides more accurate and efficient estimates of the treatment effect in the PK data by taking into account the impact of immunogenicity. The efficiency can be gained only when the PK and immunogenicity outcomes depend on different covariate sets, and the gained efficiency depends on: 1) the strength of correlation between PK and ADA data (it is a strong factor, the higher correlation strength, the more variability explained by ADA data, the more efficiency gained); 2) the strength of correlation between ADA and covariate (it is a weak factor, increasing correlation strength leads to slight increase in the power).

We exemplify the proposed method in the real data from two PK similarity clinical studies with highly immunogenic biologics. For parameters that are less impacted by immunogenicity, factorization model provides similar estimation as ANOVA, this suggests robustness of factorization model in the treatment effect estimation. For parameters that are substantially impacted by immunogenicity, factorization model provides efficient estimations. It is interesting that when the non-product-related factors impact immunogenicity and hence the PK outcome (like AVT02-GL-102 where the exact same manufacturing batch was used in both treatment groups), factorization model reduces the bias that was introduced by the influence of immunogenicity.

We encourage the use of factorization model to design and analyze PK similarity studies of highly immunogenic products when it is expected that potential difference in immunogenicity between treatment groups is due to non-product-related factors (e.g., the PK similarity studies with different devices administering the same product). For PK similarity studies using different highly immunogenic products, factorization model can be an option for sensitivity analysis when there are no clinically meaningful differences in immunogenicity profiles between treatment groups.

## Supplementary Information

**Additional file 1.**

**Additional file 2.**

## Data Availability

The datasets used and/or analysed during the current study available from the corresponding author on reasonable request.
