# Supplementary figures and images for: Joint analysis of PK and immunogenicity outcomes using factorization model − a powerful approach for PK similarity study
Source: BMC Med Res Methodol. 2022 Oct 8;22:264. doi: 10.1186/s12874-022-01742-2 (PMC9547438; doi:10.1186/s12874-022-01742-2)

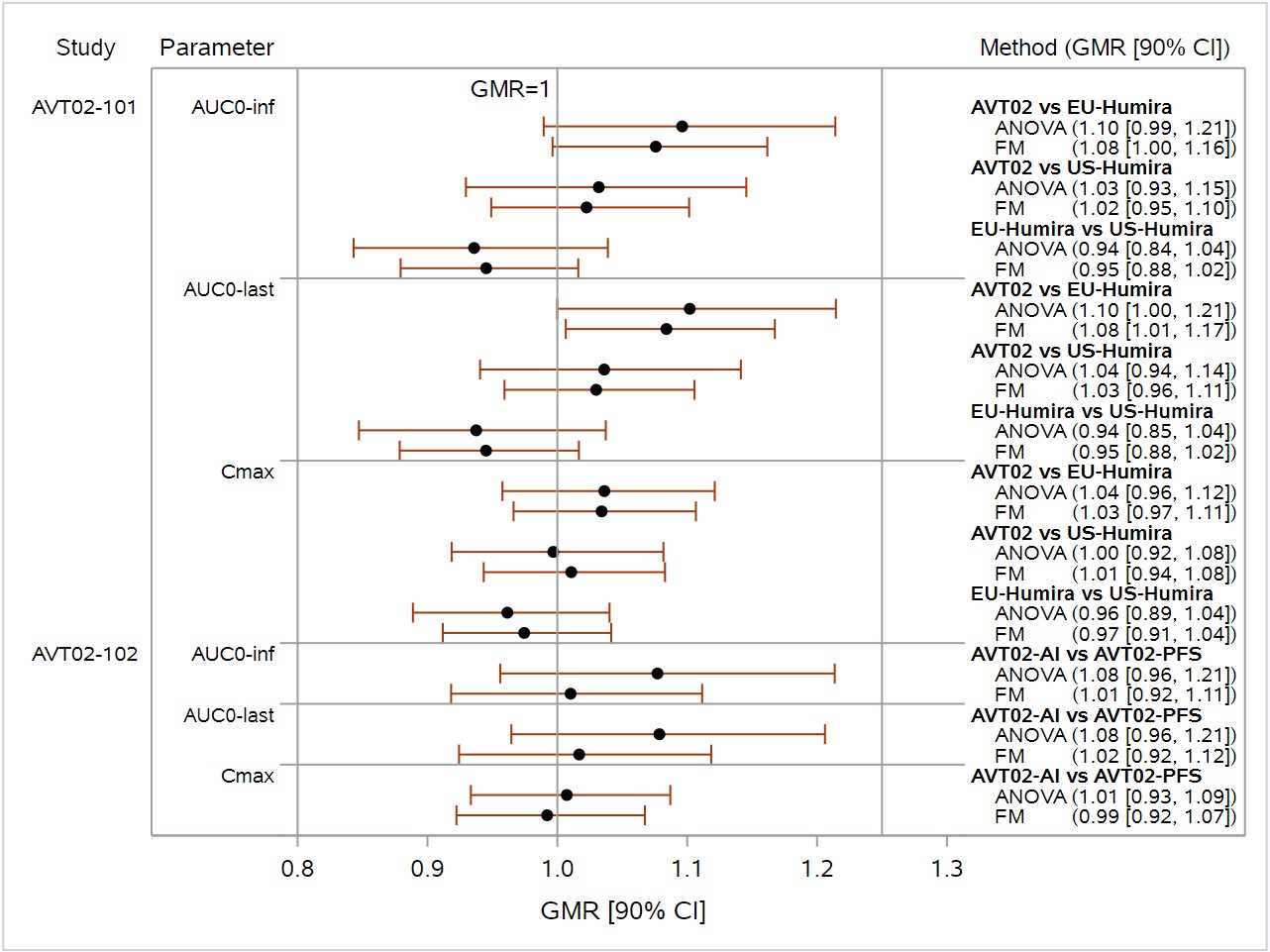

Supplement: Supplementary file 1 — Additional file 1. [file 12874_2022_1742_MOESM1_ESM.zip › Supplementary Figure A.jpg]

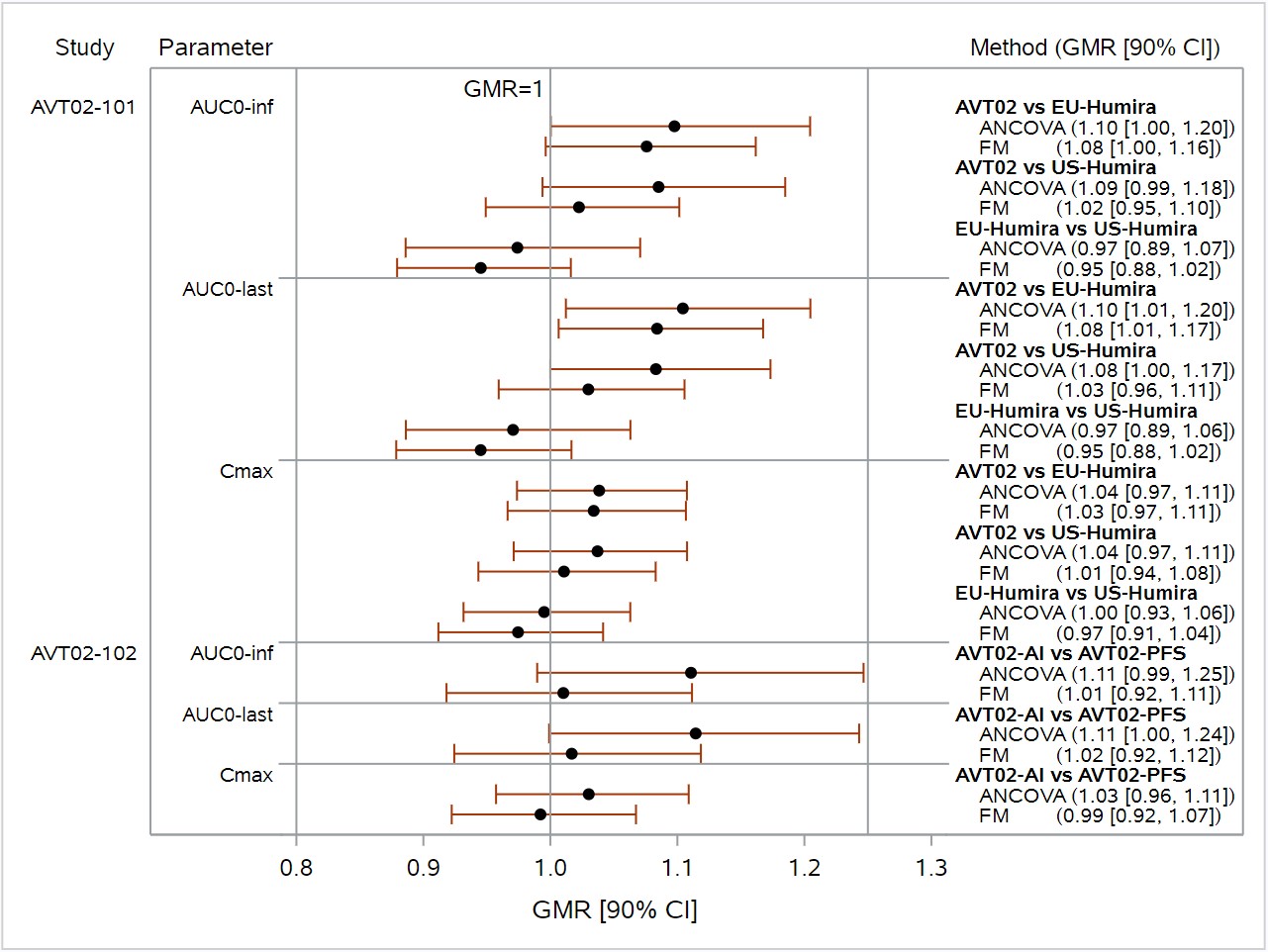

Supplement: Supplementary file 1 — Additional file 1. [file 12874_2022_1742_MOESM1_ESM.zip › Supplementary Figure B.jpg]
